# Supplementary figures and images for: Mutation of NEKL-4/NEK10 and TTLL genes suppress neuronal ciliary degeneration caused by loss of CCPP-1 deglutamylase function
Source: PLoS Genet. 2020 Oct 16;16(10):e1009052. doi: 10.1371/journal.pgen.1009052 (PMC7592914; doi:10.1371/journal.pgen.1009052)

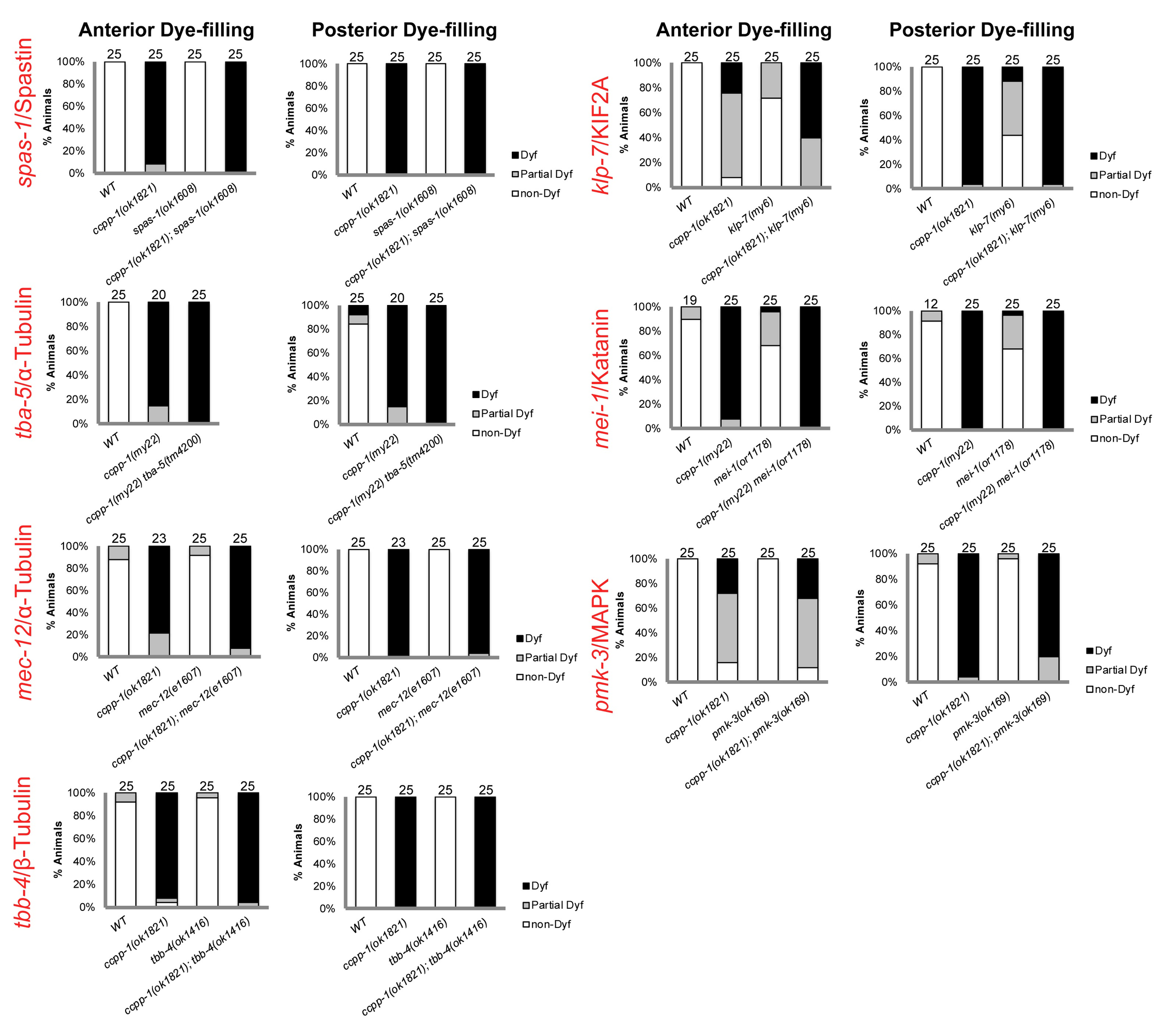

Supplement: S1 Fig — For experiments involving the mei-1(or1178) temperature-sensitive allele, eggs from plates kept at the permissive temperature (15°C; [112]) were picked to fresh plates and shifted to 25°C after hatching for 4 days before scoring. (TIF) [file pgen.1009052.s001.tif]

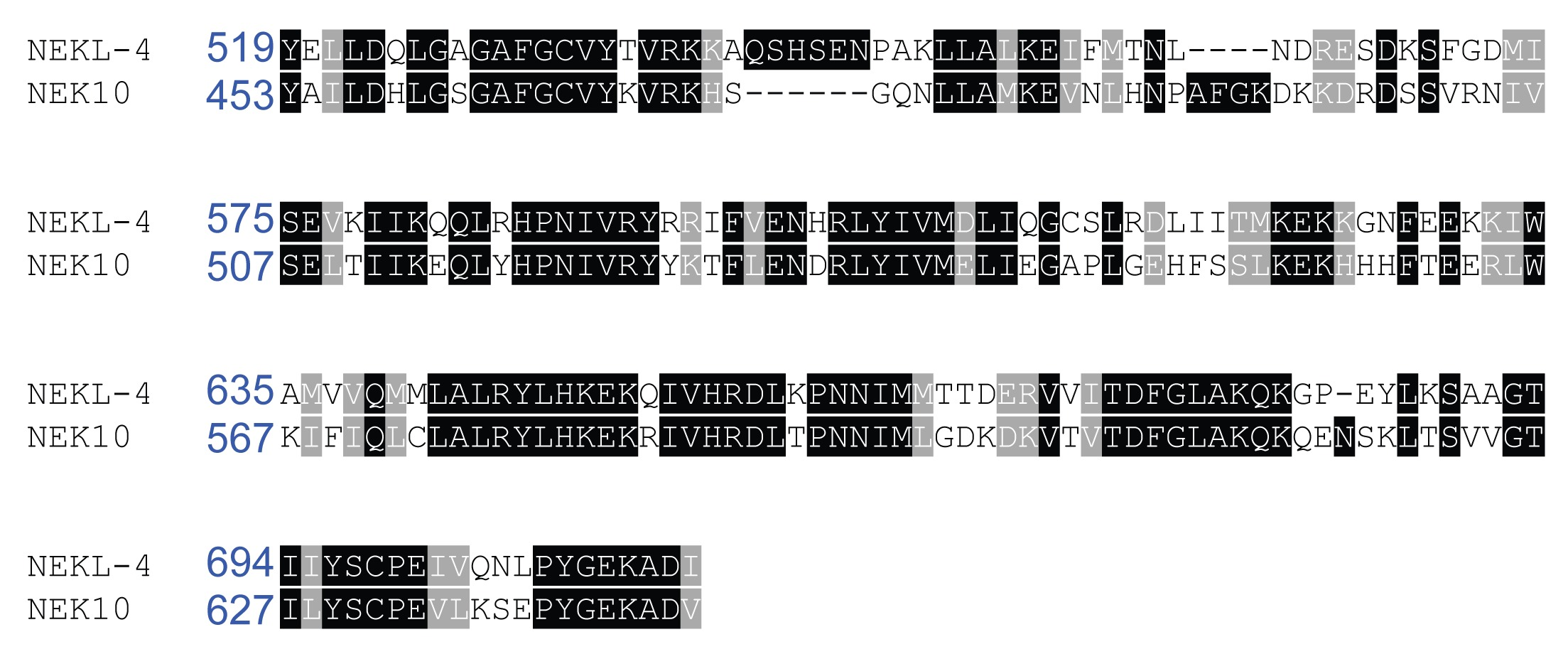

Supplement: S2 Fig — (TIF) [file pgen.1009052.s002.tif]

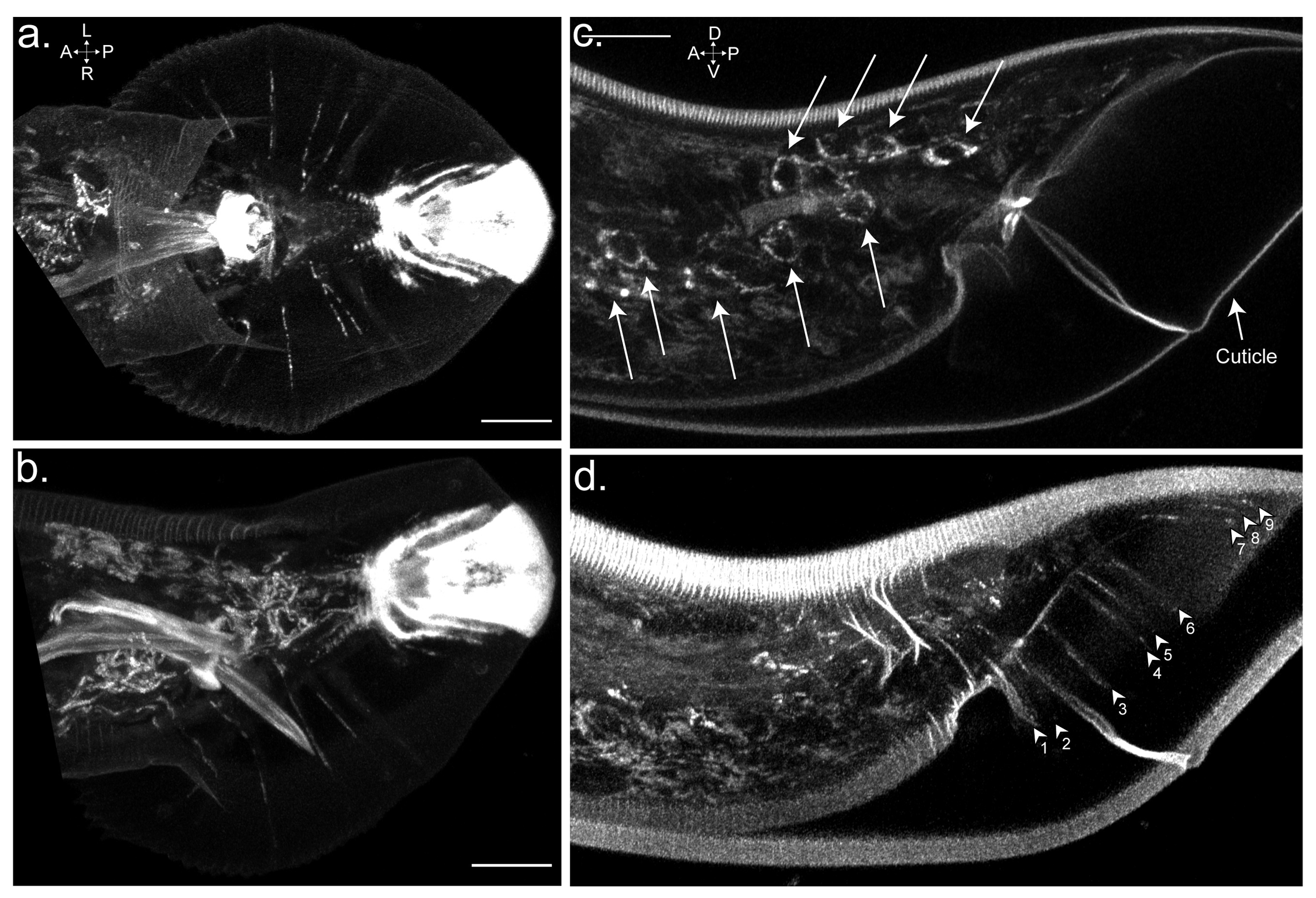

Supplement: S3 Fig — a-b. Localization of NEKL-4::mNeonGreen in adult males. Tail is flat against the coverslip in a. to visualize ray dendrites. Tail in b. is folded but the filamentous pattern in ray cell bodies is visible. c-d. Localization of NEKL-4::mNeonGreen in L4 molt males. Both images are different sections from the same z-stack. Arrows indicate ray cell bodies, arrowheads indicate ray dendrites (numbered). Scale = 10μm. (TIF) [file pgen.1009052.s003.tif]

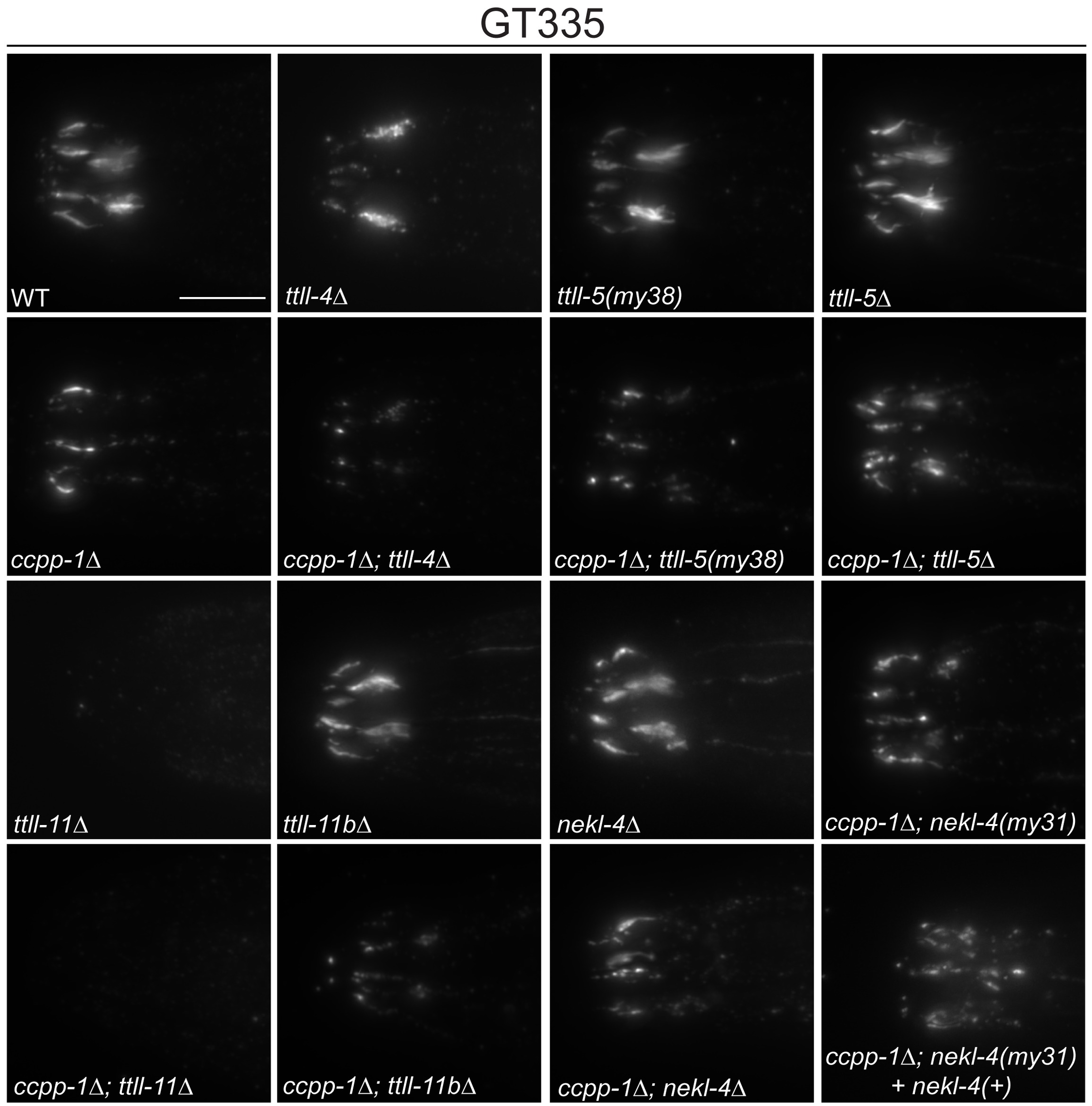

Supplement: S4 Fig — Scale bar = 10μm. (TIF) [file pgen.1009052.s004.tif]

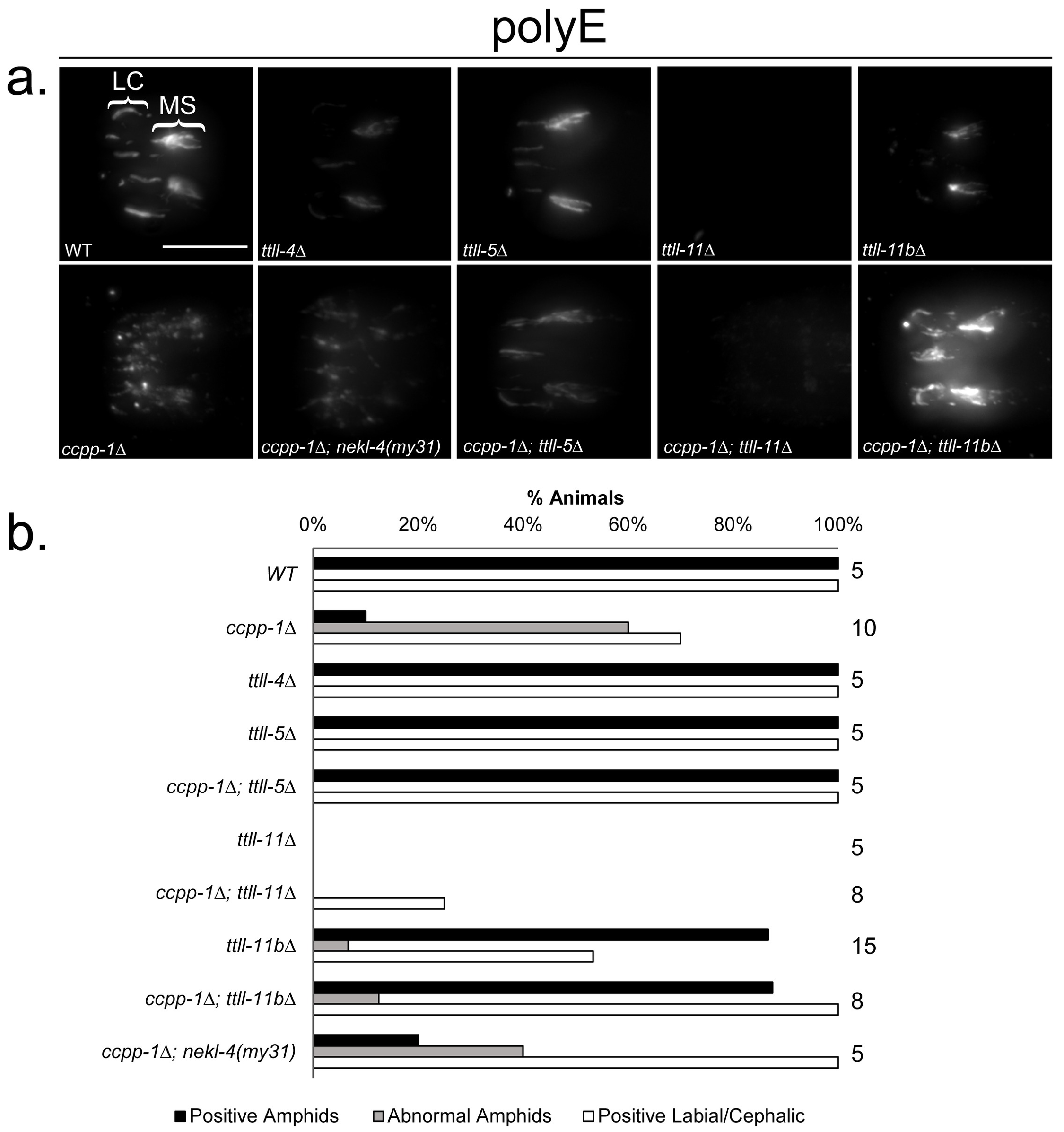

Supplement: S5 Fig — a. Example images of polyE stained cilia in the nose. The polyE polyclonal antibody stained amphid ciliary middle segments (MS) and labial and cephalic cilia (LC), similar to GT335. Abnormal polyE staining was visible in some strains containing the ccpp-1 deletion mutation. Loss of TTLL-11 abolished polyE staining, but in the absence of TTLL-4 or TTLL-5, polyE staining remained. Scale = 10μm. b. Quantification of polyE staining phenotypes by genotype; number of animals examined indicated at right. (TIF) [file pgen.1009052.s005.tif]
