# Supplementary material for: Mutation of NEKL-4/NEK10 and TTLL genes suppress neuronal ciliary degeneration caused by loss of CCPP-1 deglutamylase function
Source: PLoS Genet. 2020 Oct 16;16(10):e1009052. doi: 10.1371/journal.pgen.1009052 (PMC7592914; doi:10.1371/journal.pgen.1009052)
Supplement: S1 Reference — (DOCX) [file pgen.1009052.s013.docx]

**Supplemental References**

113. [Meissner B, Rogalski T, Viveiros R, Warner A, Plastino L, Lorch A, et al. Determining the sub-cellular localization of proteins within Caenorhabditis elegans body wall muscle. PLoS One. 2011;6: e19937. doi:](http://paperpile.com/b/eZU78c/hrozf)[10.1371/journal.pone.0019937](http://dx.doi.org/10.1371/journal.pone.0019937)

114. [Shaye DD, Greenwald I. OrthoList: a compendium of C. elegans genes with human orthologs. PLoS One. 2011;6: e20085. doi:](http://paperpile.com/b/eZU78c/Aw0SE)[10.1371/journal.pone.0020085](http://dx.doi.org/10.1371/journal.pone.0020085)

115. [Kim W, Underwood RS, Greenwald I, Shaye DD. OrthoList 2: A New Comparative Genomic Analysis of Human and Caenorhabditis elegans Genes. Genetics. 2018;210: 445–461. doi:](http://paperpile.com/b/eZU78c/jc6GR)[10.1534/genetics.118.301307](http://dx.doi.org/10.1534/genetics.118.301307)

116. [Bar-Lavan Y, Shemesh N, Dror S, Ofir R, Yeger-Lotem E, Ben-Zvi A. A Differentiation Transcription Factor Establishes Muscle-Specific Proteostasis in Caenorhabditis elegans. PLoS Genet. 2016;12: e1006531. doi:](http://paperpile.com/b/eZU78c/8bDEE)[10.1371/journal.pgen.1006531](http://dx.doi.org/10.1371/journal.pgen.1006531)

117. [Rashid S, Breckle R, Hupe M, Geisler S, Doerwald N, Neesen J. The murine Dnali1 gene encodes a flagellar protein that interacts with the cytoplasmic dynein heavy chain 1. Mol Reprod Dev. 2006;73: 784–794. doi:](http://paperpile.com/b/eZU78c/fD1pa)[10.1002/mrd.20475](http://dx.doi.org/10.1002/mrd.20475)

118. [Schou KB, Mogensen JB, Morthorst SK, Nielsen BS, Aleliunaite A, Serra-Marques A, et al. KIF13B establishes a CAV1-enriched microdomain at the ciliary transition zone to promote Sonic hedgehog signalling. Nat Commun. 2017;8: 14177. doi:](http://paperpile.com/b/eZU78c/pky84)[10.1038/ncomms14177](http://dx.doi.org/10.1038/ncomms14177)

119. [Xu Q, Zhang Y, Wei Q, Huang Y, Li Y, Ling K, et al. BBS4 and BBS5 show functional redundancy in the BBSome to regulate the degradative sorting of ciliary sensory receptors. Sci Rep. 2015;5: 11855. doi:](http://paperpile.com/b/eZU78c/d4LNV)[10.1038/srep11855](http://dx.doi.org/10.1038/srep11855)

120. [Mijalkovic J, van Krugten J, Oswald F, Acar S, Peterman EJG. Single-Molecule Turnarounds of Intraflagellar Transport at the C. elegans Ciliary Tip. Cell Rep. 2018;25: 1701–1707.e2. doi:](http://paperpile.com/b/eZU78c/99Gk7)[10.1016/j.celrep.2018.10.050](http://dx.doi.org/10.1016/j.celrep.2018.10.050)

121. [Kobayashi T, Gengyo-Ando K, Ishihara T, Katsura I, Mitani S. IFT-81 and IFT-74 are required for intraflagellar transport in C. elegans. Genes Cells. 2007;12: 593–602. doi:](http://paperpile.com/b/eZU78c/4QSi2)[10.1111/j.1365-2443.2007.01076.x](http://dx.doi.org/10.1111/j.1365-2443.2007.01076.x)

122. [Al-Jassar C, Andreeva A, Barnabas DD, McLaughlin SH, Johnson CM, Yu M, et al. The Ciliopathy-Associated Cep104 Protein Interacts with Tubulin and Nek1 Kinase. Structure. 2017;25: 146–156. doi:](http://paperpile.com/b/eZU78c/bLVw5)[10.1016/j.str.2016.11.014](http://dx.doi.org/10.1016/j.str.2016.11.014)
